# Single‐cell sequencing of mouse heart cellular heterogeneity in hypercholesterolemia reveals the mechanism of myocardial damage

**DOI:** 10.1002/ctm2.951

**Published:** 2022-07-20

**Authors:** Caihui Zhang, Yue Sun, Chen Yang, Qiuyue Wang, Yao Lu

**Affiliations:** ^1^ Department of Anesthesiology The First Affiliated Hospital of Anhui Medical University Hefei China; ^2^ Ambulatory Surgery Center The First Affiliated Hospital of Anhui Medical University Hefei China

To the Editor,

Cardiovascular diseases are a leading cause of death worldwide, accounting for approximately one‐third of all deaths.^1^ As a major risk factor for cardiovascular disease, hypercholesterolemia causes direct myocardial damage. Previous studies have shown that hypercholesterolemia may cause myocardial reactive oxidative stress and mitochondrial dysfunction, ultimately resulting in myocardial damage.^2^, ^3^ Nevertheless, there is still limited information on the specific molecular mechanisms that underlie this condition. Inflammation is a key process in cardiovascular diseases associated with hypercholesterolemia, such as atherosclerosis, and includes the activation of T lymphocytes.^4^ Single‐cell RNA sequencing is an essential research method for detecting the cellular changes and molecular processes of the heart.^5^ Currently, single‐cell sequencing studies in hypercholesterolemia have mainly focused on the changes in inflammatory cell subtypes in atherosclerotic plaques,^6^ and the changes in the cellular composition in the heart remain unknown. Therefore, we used single‐cell sequencing to detect all heart cells in the early and late stages of disease to explore the molecular mechanism of myocardial damage.

First, we utilized Apoe^−/−^ mice fed a high‐cholesterol diet to establish a hypercholesterolemia model. In the process, the mouse serum lipoprotein index of TC and LDL increased significantly, and HE and ORO staining indicated that lipid levels increased (Figure S1A–E). Then, we identified 12 cell clusters of heart tissues according to the marker genes via single‐cell sequencing (Figure 1A–D). When ordering these clusters according to abundance, the most abundant cell population was fibroblasts. Inflammatory cells, such as myeloid cells, B cells and T cells, increased in the disease group (Figure 1E).

**FIGURE 1:**
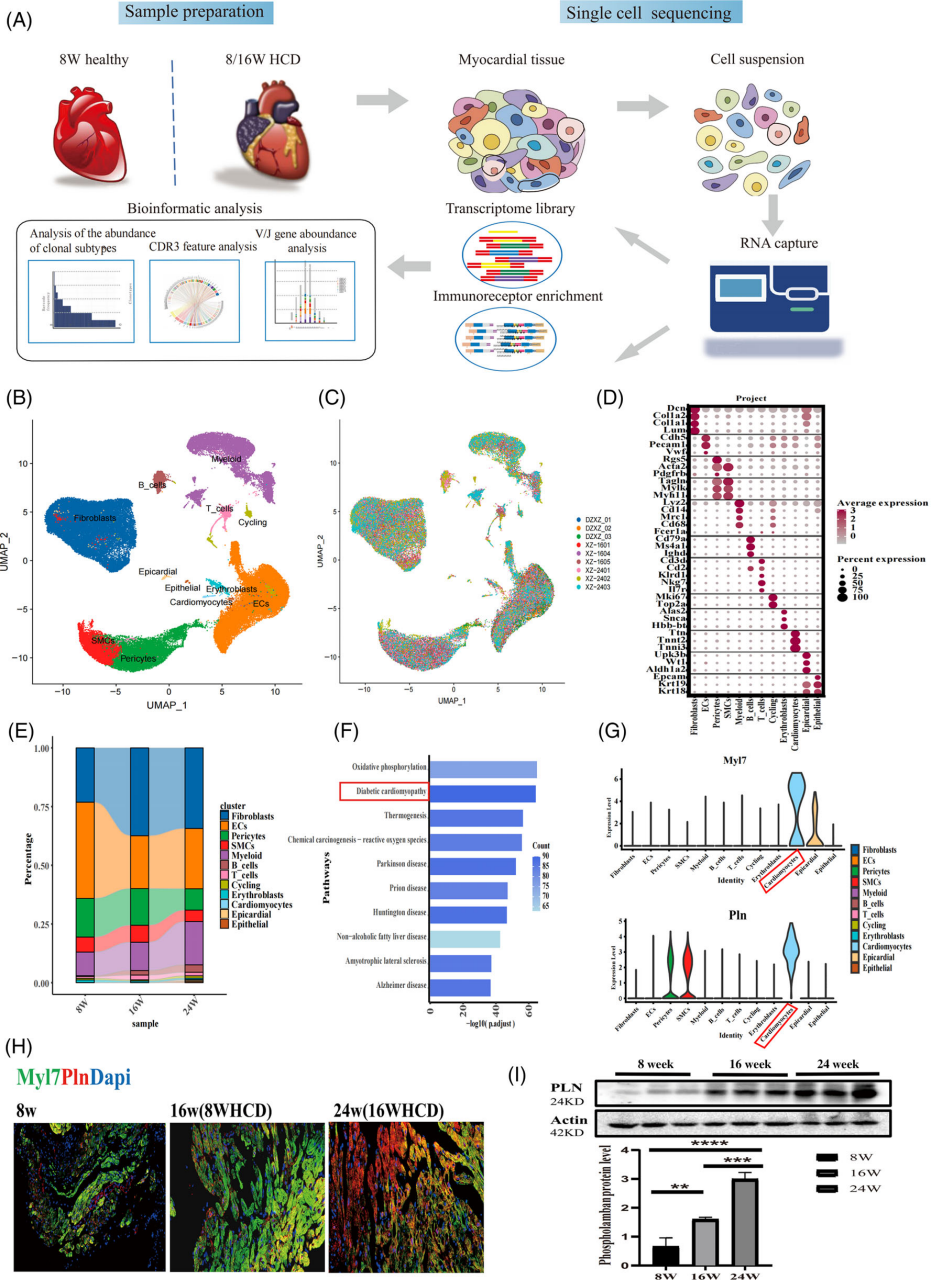
Overview of the cell composition of the mice heart. (A) Schematic of the study design and workflow. Heart tissues dissociation and single‐cell suspension preparation. Cells isolated from the heart tissue were selected for subsequent experiments. (B) Two‐dimensional *t*‐distributed stochastic neighbour embedding (*t*‐SNE) visualization of 84 696 cardiac cells identified 12 different clusters after unsupervised clustering. Each point depicts a single cell, coloured according to cluster designation. (C) *t*‐SNE projection of all cardiac cell populations (as in B) coloured by experimental condition (dark blue, orange and dark green: 8‐week mice (baseline, *n* = 3 mice), red, purple and brownness: 16‐week mice (8‐week HCD, *n* = 3 mice), pink, light green and light blue: 24‐week mice (16‐week HCD, *n* = 3 mice). (D) Dotplot showing the top 10 most differentially expressed genes in each cluster identified through unsupervised clustering of cardiac cells, the depth of the red colour represents the level of genes’ average expression, and the circular size indicates the genes’ expression proportion in the cluster. (E) Bar plot showing the proportions of cells in each of the 12 identified cell populations, coloured according to cluster designation. Identified cell types are shown on the right. (F) Gene ontology (GO) enrichment analysis of cardiomyocyte. The longer the column, the more significant the enrichment result. (G) Expression distribution (violin plot) showing normalized expression levels of cardiomyocyte genes Myl7 and Pln signature in all identified clusters. (H) Immunofluorescence co‐staining of Myl7 and Pln, scale bars, 50 μm. (I) The protein expression of Pln in the hearts of mice was determined by Western blotting. *n* = 6 per group. Data are presented as mean ± SD. ****p < .001, ***p < .001, **p < .01, and *p < .05 represent significant differences in the 8‐, 16‐ (8‐week HCD) and 24‐week (16‐week HCD) group.

Next, cardiomyocytes were identified based on the cardiomyocyte marker genes Ttn and Tnnt2 (Figure 1D). Cardiomyocytes highly expressed the atria‐specific genes Sln, Nppa, My17 and Myh6, suggesting that they were associated with atrial function^7^ (Table S4). Additionally, cardiomyocytes were mainly distributed in disease samples (Figure 1E). Therefore, we performed Kyoto Encyclopedia of Genes and Genomes (KEGG) analysis to further investigate cardiomyocyte function. Interestingly, there was significant enrichment of genes associated with diabetic cardiomyopathy (DCM) in cardiomyocytes, so we selected the cardiomyocyte‐specific related gene Myl7 and the DCM‐related gene Pln to verify the changes in these cell clusters by immunofluorescence colocation analysis (Figures 1F,G and S1F). The results suggested that Myl7 and Pln expression and the number of cardiomyocyte clusters were increased, and the Pln protein levels were also significantly increased (Figure 1H,I).

Then, we mapped the immune microenvironment in the heart under hypercholesterolemia. T cells demonstrated significant changes in the differentially expressed gene analysis, so they were further subdivided into six subpopulations. The changes in the abundance of proliferating T cells and Tn cells showed the opposite trend, which might indicate an activated differentiation process (Figure 2A–E). Trajectory analysis showed that naive cells differentiated into two major branches, Th cells and proliferating populations (Figures 2F and S2A). The proportion of Th cells and CD8+ T cells increased significantly during treatment with a high‐cholesterol diet; this was especially true for Th cells (Figure 2C). Immunofluorescence staining for the specific markers T and Th cells, CD3 and CD4, also showed that Th cells were significantly infiltrated in the disease group (Figure 2H).^8^ The Il‐17a, Ccr6 and Cd163l1 genes were highly expressed, illustrating that the Th‐cell subpopulation was Th17 cells (Figure 2G). Immunohistochemical results revealed obvious positivity for IL‐17, revealing that Th17 cells mainly accumulated in  the heart tissues of mice fed an HCD for 8 and 16 weeks. Additionally, IL‐17a is an important cytokine secreted by Th17 cells, and the ELISA and WB results from tissue homogenates showed that IL‐17a and IL‐17‐related proteins were significantly increased in our study (Figure 2I–K).

**FIGURE 2:**
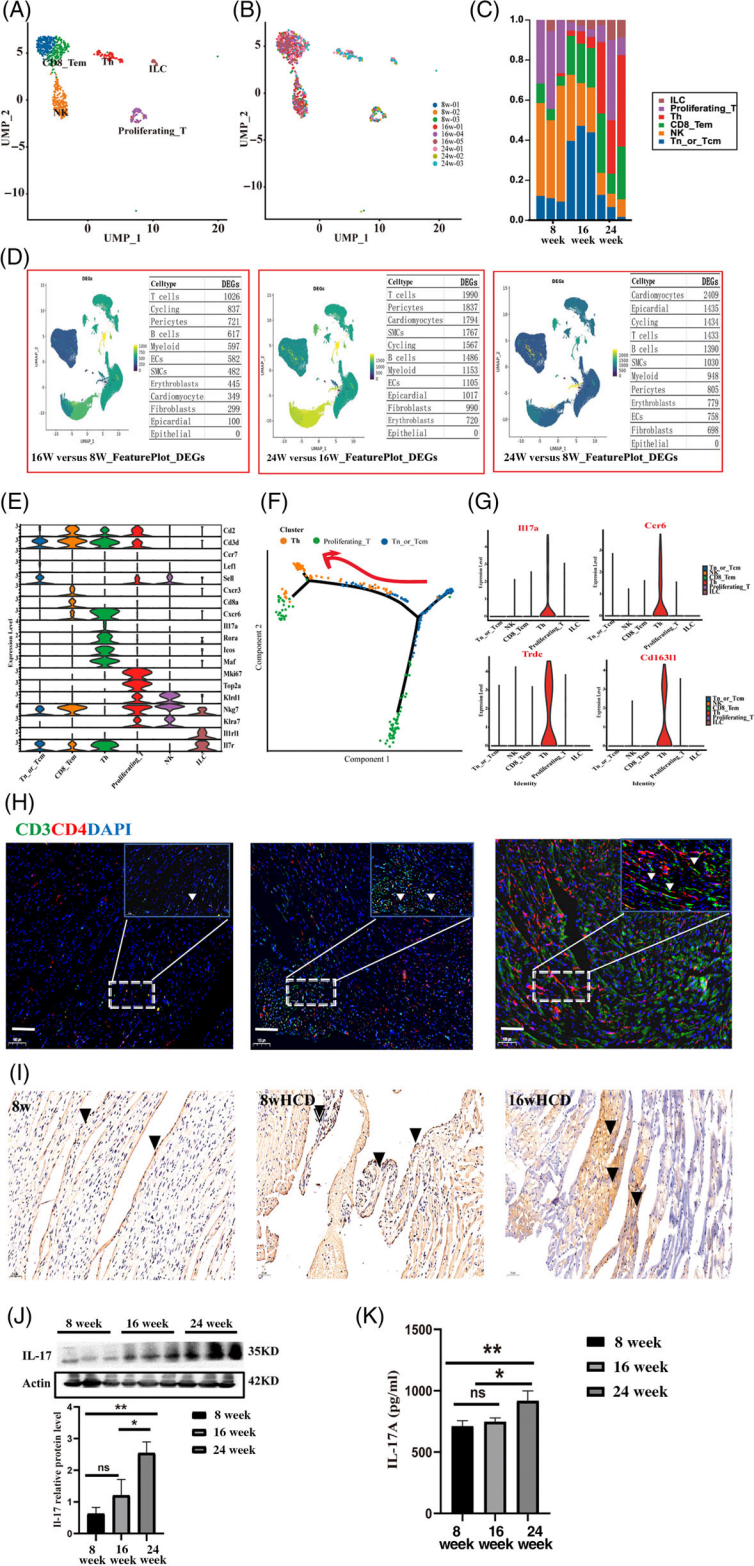
Hypercholesterolemia induces the expansion of T cells. (A and B) *t*‐SNE projection of the 6 clusters identified in the heart, or sample source (B). (C) Bar plot showing the proportions of cells in each of the six identified cell populations. (D) Heat map showing differentially expressed genes (DEGs) in each cell type. Source data are available online. (E) The violin diagram represents the expression of each marker gene in each T‐cell cluster. The vertical represents the genes expression level, and the horizontal represents the density distribution under the expression value. (F) The ordering of subpopulations of T cells along pseudotime in a two‐dimensional state‐space defined by Monocle2. Each point corresponds to a single cell, and each colour represents a type of cell cluster. (G) Expression distribution (violin plot) showing normalized expression levels of Th cells genes Il‐17a, Ccr6 and Cd163l1 signature in all identified clusters. (H) Immunofluorescence co‐staining of CD4 and CD3, scale bars, 100 μm (left), 50 μm (top right corner). (I) The expression of IL‐17 was detected by immunohistochemistry. The scale bar represents 50 μm. (J) The protein expression of IL‐17 in the hearts of mice was determined by Western blotting. (K) Tissue homogenate levels of IL‐17 were determined by ELISA. *n* = 6 per group. Data are presented as mean ± SD. ****p < .001, ***p < .001, **p < .01 and *p < .05 represent significant differences in the 8‐, 16‐ (8‐week HCD) and 24‐weeks (16‐week HCD) group.

Additionally, fibroblasts were further subdivided into seven subpopulations (Figure 3A,B). The proportion of Cluster 2 was increased as the disease (Figure C, Table S10). According to the gene ontology (GO)/KEGG analysis of Cluster 2, inflammatory genes and the IL‐17 signalling pathway were obviously enriched (Figure 3D,E). Previous studies suggested that the IL‐17 signalling pathway is related to myocardial fibrosis.^9^ Our results revealed that fibrosis was more serious in the disease groups, as shown in Figure 3G. We confirmed the source of IL‐17 via immunofluorescence staining. The results showed that the majority of IL‐17‐positive cells were vimentin+ fibroblasts, except in rare cases where α‐actinin + cardiomyocytes and rarely costained with CD31. These findings suggest that IL‐17 mainly originates from fibroblasts, which is consistent with a prior study (Figure 3G).^10^ The genes and pathways that were enriched in fibroblast Cluster 3 were associated with collagen deposition (Figure 3H,I). Sirius staining confirmed this increase in collagen content. Type I and III collagen fibres were obviously deposited, and the collagen III protein expression level in fluorescence localization was higher in the 8/16‐week HCD groups (Figure 3J,K). Moreover, GO/KEGG analysis of Cluster 6 suggested that fibrosis‐related genes and the Wnt pathway were enriched, and the levels of wnt5a and β‐catenin proteins, which are key mediators of the Wnt‐signalling pathway, were significantly increased in cardiac tissues (Figures 3F,L and S1G,H).

**FIGURE 3:**
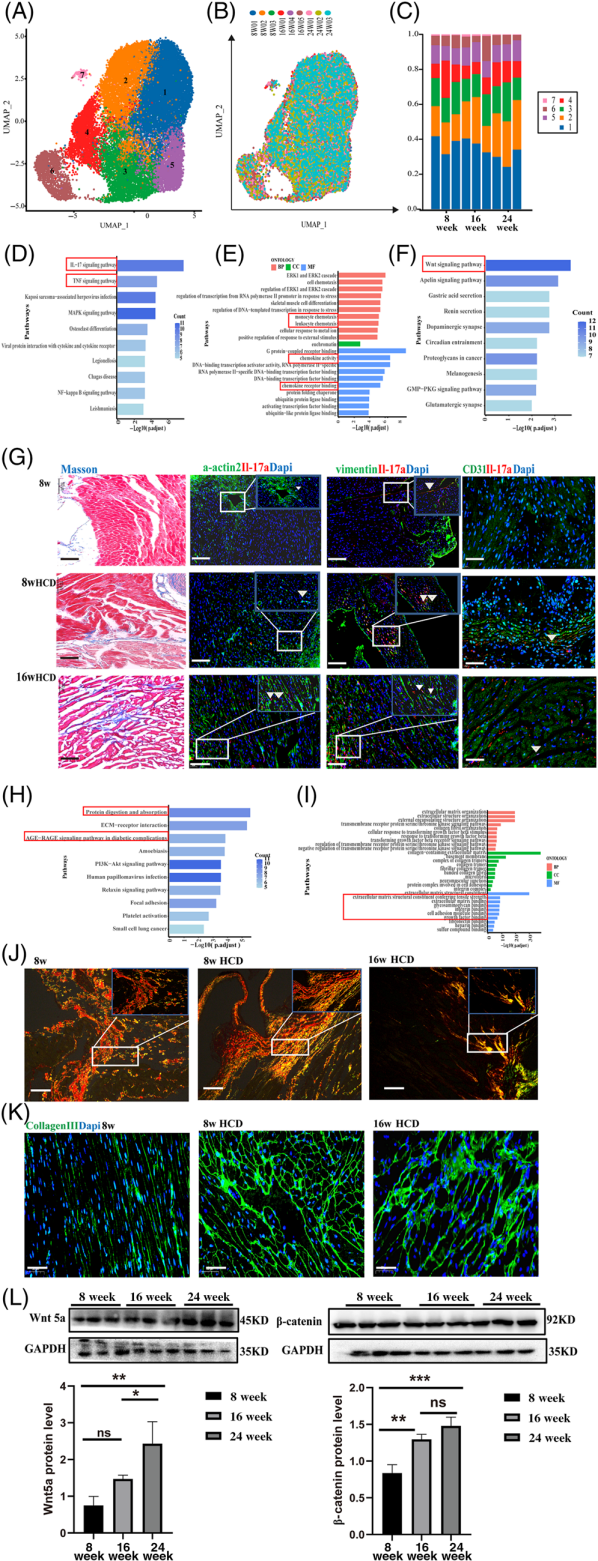
Hypercholesterolemia resulted in fibrosis and collagen deposition in mice heart. (A and B) *t*‐SNE projection of the seven clusters identified in the heart, or sample source (B). (C) Bar plot showing the proportions of cells in each of the seven identified cell populations. (D–F) Gene ontology (GO) and Kyoto Encyclopedia of Genes and Genomes (KEGG) enrichment analysis of the fibroblast Cluster 2. KEGG enrichment analysis of fibroblast Cluster 6 (F), the longer the column, the more significant the enrichment result. (G) Representative images of Masson‐stained of heart sections. The image of blue is collagen fibre, and the scale bar represents 100 μm. The immunofluorescence co‐staining of α‐actin2 and Il‐17a, vimentin and Il‐17a, CD31 and Il‐17a. Scale bars, 50 μm (left), 25 μm (top right corner). (H and I) GO and KEGG enrichment analysis of Cluster 3 of fibroblast. (J) Representative polarization microscopy images of sirius red stained sections. Yellow is collagen fibre Ⅰ, and green is collagen fibres III. Scale bars, 50 μm (left), 25 μm (top right corner) (K), the immunofluorescence staining of collagen III, scale bars, 50 μm. (L) The protein expression of wnt5a and β‐catenin in the hearts of mice was determined by Western blotting. *n* = 6 per group. Data are presented as mean ± SD. ****p < .001, ***p < .001, **p < .01 and *p < .05 represent significant differences in the 8‐, 16‐ (8‐week HCD) and 24‐week (16‐week HCD) group.

Next, we further performed clustering analysis for macrophages. Macrophages are a subtype of myeloid cells, and they accounted for the largest proportion of this cell type (Figure 4A–C). Macrophages were subdivided into six subpopulations via marker genes. Among these subpopulations, Cluster 1 was the most abundant, and Cluster 2 showed an increasing trend (Figure 4D–F). Moreover, the enrichment analysis of Cluster 1 showed that leukocyte migration and chemotaxis were obviously enriched, and the function of Cluster 2 was mainly related to the apoptotic pathway (Figures 4G and S2B). Macrophages influence T‐cell activation by regulating MHC‐II and may affect fibroblasts through TGF‐β pathways (Figures 4H,I and S2E,F). In CellChat analysis, T cells, fibroblasts and macrophages had significant and obvious interactions (Table S8), and CXCL12‐CXCR4 and CCL5‐CCR5 were significant chemokine interactions among them (Figure 4J,K). Additionally, the smooth muscle cell subpopulation Cluster 5 also expressed the fibroblast markers Dcn and Lum and was thus determined to be myofibroblasts (Table S17). Lymphatic endothelial cells, plasma cells and activated B cells were mainly distributed in disease samples, which may indicate the activation of inflammatory responses (Figure S3A–F).

**FIGURE 4:**
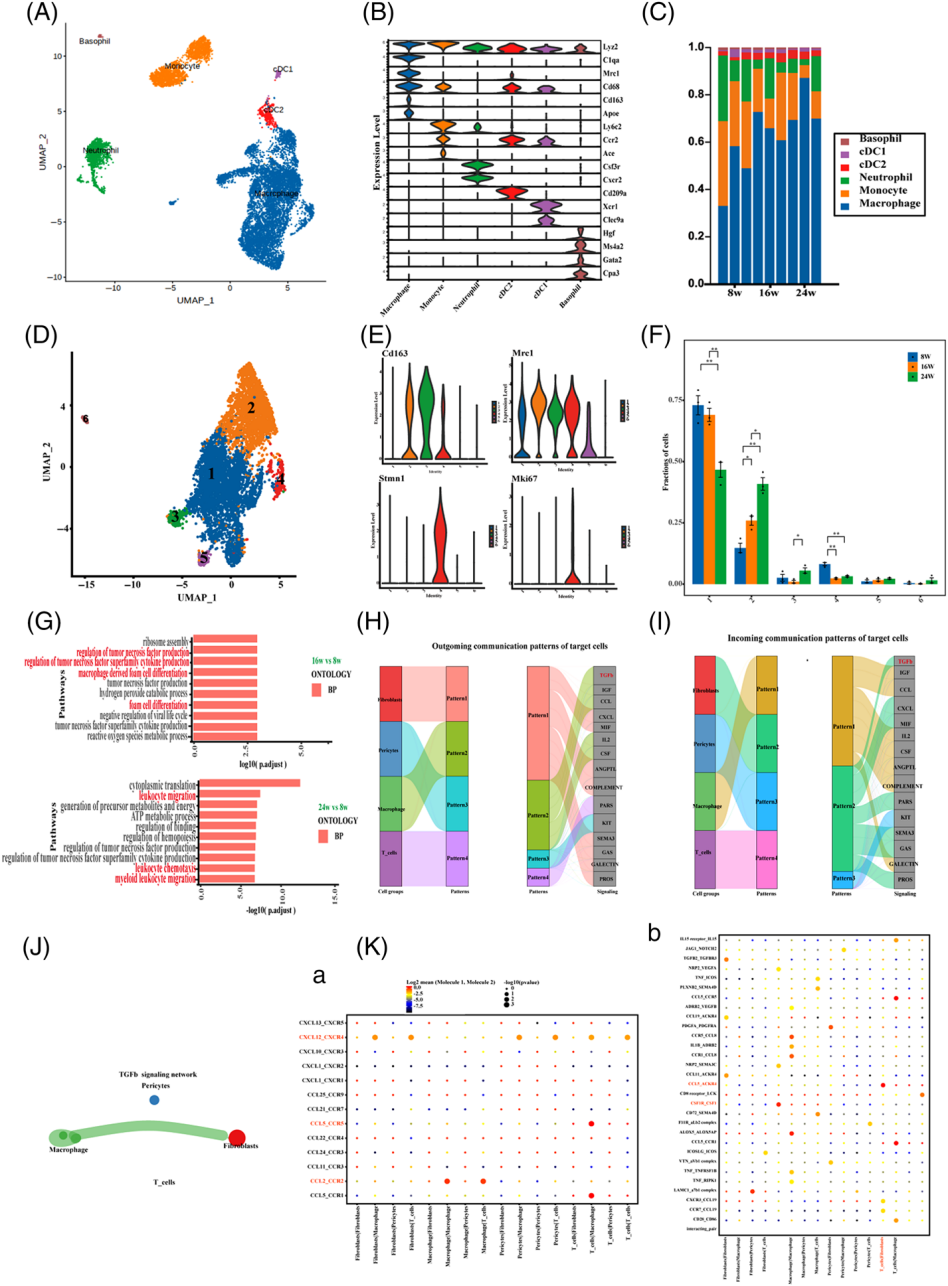
Cardiac macrophages and myeloid cells. (A and D) *t*‐SNE projection of the subpopulation of macrophages and myeloid cells identified in the heart. (C) Bar plot showing the proportions of cells in each of the subpopulation of myeloid cells. (B) The violin diagram represents the expression of marker gene in each subpopulation of myeloid cells. (E) The violin diagram represents the expression of marker gene in the subpopulation of macrophages. The subpopulations of macrophages expressed marker genes Mrc1 and Cd163 of M2‐type macrophages. Cluster 4 was proliferating M2 macrophage, expressing proliferation‐related marker genes Mki67 and Stmn1. (F) The horizontal axis represents each subpopulation of macrophages, the vertical axis represents the average proportion of each cell type to the total number of each entire sample, and the colour represents the sample group (dark blue: 8‐week mice [baseline], orange: 16‐week mice (8‐week HCD), dark green: 24‐week mice (16‐week HCD). (G) Gene ontology (GO) enrichment analysis of the cell Cluster 1 of macrophages in each group, (above: 16 vs. 8 weeks, 24 vs. 8 weeks). (H and I) Fibroblast and macrophages populations operate in coordination with signalling TGF‐β pathways. The figure on the left shows four cell types divided into four patterns, and the figure on the left shows the key output signals of each pattern (H) and the key incoming signals of each pattern (I). (J) Network diagram of interaction between all cell types, in which network nodes are T cell, fibroblast, macrophages and pericyte. Network edge thickness is the total number of ligand and receptor pairs, and line colour is consistent with ligand cell type. (K), a, Chemokine interaction bubble diagram; b, the diagram shows the top 30 ligand pairs between the two cell types. The size of the circle represents the significance of the interaction, and the face of the circle color represents interaction strength (gene expression).

In summary, hypercholesterolemia is critical in cardiovascular diseases, and there is an urgent need to develop effective therapeutics. The high expression of Pln genes in cardiomyocytes may provide insight into the possible causes of myocardial injury. The interaction among macrophages, T cells, fibroblasts and collagen deposition emphasize the importance of fibrosis and inflammatory activation. This interaction might partly explain the mechanisms of hypercholesterolemia and provide treatment strategies for cardiovascular diseases.

## CONFLICT OF INTERESTS

The authors declare that there is no conflict of interest.

## Supporting information

Supporting Information

Figure S1

Figure S2

Figure S3

Table S1

Table S2

Table S3

Table S4

Table S5

Table S6

Table S7

Table S8

Table S9

Table S10

Table S11

Table S12

Table S13

Table S14

Table S15

Table S16

Table S17
