# Supplementary material for: Single‐cell sequencing of mouse heart cellular heterogeneity in hypercholesterolemia reveals the mechanism of myocardial damage
Source: Clin Transl Med. 2022 Jul 20;12(7):e951. doi: 10.1002/ctm2.951 (PMC9301086; doi:10.1002/ctm2.951)
Supplement: Supplementary file 1 — Supporting Information [file CTM2-12-e951-s004.doc]

**Single Cell Sequencing Methods and Materials**

## Sample collection and processing

After anesthesia of mice in different treatment stages, heart tissue was taken for subsequent experiments.

## Tissue dissociation and single-cell suspension preparation

The heart tissues were conserved in the GEXSCOPE Tissue Preservation Solution (Singleron) and shipped to the Singleron lab with ice pack. The specimens were washed 3 times with Hanks Balanced Salt Solution (HBSS，Gibco, Cat. No.14025-076) and shred into 1–2 mm pieces. Then the tissue debris were submitted to the digestion with 2ml GEXSCOPE Tissue Dissociation Solution (Singleron) at 37℃ for 15min in 15ml centrifuge tube （Falcon, Cat. No.352095）with sustained agitation. Cells were filtered through 40-micron sterile strainers（Falcon, Cat. No.352340）to and centrifuged (eppendorf, 5810R)at 300g for 5 minutes. Then the supernatant was removed, and the pellets were resuspended in 1ml PBS (Hyclone, Cat. No.SA30256.01 ). To remove the red blood cells, which were frequently a signifcant portion of the cells produced, 2 mL RBC lysis buﬀer (Roche, Cat. No. 11 814 389 001) was added to the cell suspension according to the manufacturer’s protocol. Centrifuge the cells at 500 × g for 5 min in a microfuge at 15-25°C and resuspend in PBS (Hyclone, Cat. No.SA30256.01 ). The sample from the cell mixture was stained with trypan blue (Bio-RAD, Cat. No.#1450013) and microscopically(Nikon, ECLIPSE Ts2) cell count to make the concentration was 1×10^5^ cells/mL，and once the cell viability exceeded 80%, subsequent sample processing could be performed.

## Single-cell RNA-seq details and preliminary results

Single-cell suspensions with 1×10^5^ cells/mL in concentration in PBS were prepared. Single-cell suspensions were then loaded into microfluidic devices and scRNA-seq libraries were constructed according to Singleron GEXSCOPE protocol by GEXSCOPE Single-Cell RNA Library Kit (Singleron Biotechnologies)，which included cell lysis, mRNA trapping, labeling cells (barcode) and mRNA (UMI), reverse transcription mRNA into cDNA and amplification, and finally fragment cDNA.Individual libraries were diluted to 4 nM and pooled for sequencing. Pools were sequenced on an Illumina HiSeq X with 150 bp paired end reads. Raw reads were processed with fastQC and fastp to remove low quality reads. Poly-A tails and adaptor sequences were removed by cutadapt. After quality control, reads were mapped to the reference genome GRCh38 (ensembl version 92 annotation) using STAR. Gene counts and UMI counts were acquired by featureCounts software. Expression matrix files for subsequent analyses were generated based on gene counts and UMI counts.

## Library preparation and scRNA-seq

luidic Single-cell suspensions (1×10^5^ cells/ml) with PBS (HyClone) were loaded onto microfdevices using the Singleron Matrix® Single Cell Processing System (Singleron). Subsequently, the scRNA-seq libraries were constructed according to the protocol of the GEXSCOPE® Single Cell RNA Library Kits (Singleron)^1^. Individual libraries were diluted to 4 nM and pooled for sequencing. At last, pools were sequenced on Illumina HiSeq X with 150 bp paired end reads.

## Primary analysis of raw read data

Raw reads from scRNA-seq were processed to generate gene expression matrixes using CeleScope v1.5.2 (Singleron Biotechnologies) with default parameters. Briefly, raw reads were first processed with fastQC^2^ v0.11.4 ([https://www](https://www/). bioinformatics. babraham.ac.uk/projects/fastqc/) and fastp^3^ to remove low quality reads, and with cutadapt^4^ to trim poly-A tail and adapter sequences. Cell barcode and UMI were extracted rom R1 reads and corrected. After that, we used STAR^5^ (v2.6.1b) to map reasds to the reference genome GRCh38 (mm10). UMI counts and gene counts of each cell were acquired with FeatureCounts^6^ (v2.0.1) software. Successfully Assigned Reads with the same cell barcode, UMI and gene were grouped together to generate the gene expression matrix for further analysis.

## Quality control, dimension-reduction and clustering

Seurat v 3.1.2^7^ was used for quality control, dimensionality reduction and clustering. For each sample dataset, we filtered expression matrix by the following criteria: 1) cells with gene count less than 200 or with top 2% gene count were excluded; 2) cells with top 2% UMI count were excluded; 3) genes expressed in less than 5 cells were excluded. To reduce the influence derived from contamination and doublet in following analysis, DecontX^8^ was used to estimate and remove contamination, and DoubletFinder^9^ was used to identify and remove doublet. After filtering, 73774 cells were retained for the downstream analyses, with on average 1199 genes and 2900 UMIs per cell. Gene expression matrix was normalized and scaled using functions NormalizeData and ScaleData. Top 2000 variable genes were selected by FindVariableFeatures for PCA analysis. Batch effect between samples was removed by Harmony v1.0^10^ using the top 20 principal components from PCA. Cells were separated into clusters by FindClusters, using the top 20 adjusted principal components and resolution parameter at 1.0. Cell clusters were visualized using Uniform Manifold Approximation and Projection (UMAP) with Seurat functions RunUMAP.

## Celltype annotation

The cell type identification of each cluster was determined according to the expression of canonical markers from the reference database SynEcoSysTM (Singleron Biotechnology). SynEcoSysTM. The canonical markers and their corresponding cell types were listed in Table x.

## Differentially expressed genes (DEGs) analysis

Differentially expressed genes (DEGs) between different samples or consecutive clusters were identified with function FindMarkers based on Wilcoxon rank sum test with default parameters, and selected the genes expressed in more than 10% of the cells in both of the compared groups of cells and with an average log (Fold Change) value greater than 0.25 as DEGs. Adjusted p value was calculated by Bonferroni Correction and the value 0.05 was used as the criterion to evaluate the statistical significance. GO function enrichment analysis was carried out on the gene set using the clusterProfiler software to explore biological functions or pathways, which are significantly associated with the specifically expressed genes^11^.

## Pathway enrichment analysis

To investigate the potential functions of DEGs, the Gene Ontology (GO) and Kyoto Encyclopedia of Genes and Genomes (KEGG) analysis were used with the “clusterProfiler” R package^11^. Pathways with p_adj value less than 0.05 were considered as significantly enriched. Gene Ontology gene sets including molecular function (MF), biological process (BP), and cellular component (CC) categories were used as reference. Protein-protein interactions (PPI) of DEGs in each cluster were predicted based on known interactions of genes with relevant GO terms in the StringDB v1.22.0.

## Gene Regulatory Network Inference

To analyze transcription factor regulatory networks, we performed SCENIC R toolkit^12^ using scRNA expression matrix and transcription factors in AnimalTFDB. Regulatory networks were predicted by the GENIE3 package based on the co-expression of regulators and targets. We used the RcisTarget package to search for transcription factor binding motifs in the data. Genes involved in a predicted regulatory network were defined as a gene set, whose auc value was calculated by the AUCell package to assess the activity of the regulatory network in cells.

## Trajectory analysis

Cell differentiation trajectory was reconstructed with the Monocle2^13^. Differentially expressed genes were used to sort cells in order of spatial‐temporal differentiation. We used DDRTree to perform FindVairableFeatures and dimension-reduction. Finally, The trajectory was visualized by plot_cell_trajectory function.

## Cell-cell interaction analysis

The cell-cell interaction analysis was performed by CellPhoneDB^14^ based on receptor–ligand interactions between two cell types/subtypes. Cluster labels of all cells were randomly permuted for 1000 times to calculate the null distribution of average ligand-receptor expression levels of the interacting clusters. Individual ligand or receptor expression was thresholded with a cutoff value based on the average log gene expression distribution for all genes across all the cell types. The significant cell-cell interactions were defined as p value < 0.05 and average log expression > 0.1, which were visualized with the circlize (0.4.10) R package.

## Cell Chat analysis

CellChat (version 0.0.2) was used to analyze the intercellular communication networks from scRNA-seq data. A CellChat object was created using the R package process. Cell information was added into the meta slot of the object. The ligand-receptor interaction database was set, and the matching receptor inference calculation was performed.

## REFERENCES

1. Dura B, Choi JY, Zhang K, et al. scFTD-seq: freeze-thaw lysis based, portable approach toward highly distributed single-cell 3' mRNA profiling. *Nucleic Acids Res*. 2019;47:e16.

2. de Sena BG, Smith AD. Falco: high-speed FastQC emulation for quality control of sequencing data. *F1000Res*. 2019;8:1874.

3. Chen S, Zhou Y, Chen Y, Gu J. fastp: an ultra-fast all-in-one FASTQ preprocessor. *Bioinformatics*. 2018;34:i884-i890.

4. Martin M. Cutadapt removes adapter sequences from high-throughput sequencing reads. *Embnet Journal*. 2011;17.

5. Dobin A, Davis CA, Schlesinger F, et al. STAR: ultrafast universal RNA-seq aligner. *Bioinformatics*. 2013;29:15-21.

6. Liao Y, Smyth GK, Shi W. featureCounts: an efficient general purpose program for assigning sequence reads to genomic features. *Bioinformatics*. 2014;30:923-930.

7. Stuart T, Butler A, Hoffman P, et al. Comprehensive Integration of Single-Cell Data. *Cell*. 2019;177:1888-1902.

8. Yang S, Corbett SE, Koga Y, et al. Decontamination of ambient RNA in single-cell RNA-seq with DecontX. *Genome Biol*. 2020;21:57.

9. Mcginnis CS, Murrow LM, Gartner ZJ. DoubletFinder: Doublet Detection in Single-Cell RNA Sequencing Data Using Artificial Nearest Neighbors. *Cell Syst*. 2019;8:329-337.

10. Korsunsky I, Millard N, Fan J, et al. Fast, sensitive and accurate integration of single-cell data with Harmony. *Nat Methods*. 2019;16:1289-1296.

11. Yu G, Wang L, Han Y, He Q. clusterProfiler: an R Package for Comparing Biological Themes Among Gene Clusters. *OMICS: A Journal of Integrative Biology*. 2012;16:284-287.

12. Aibar S, Gonzalez-Blas CB, Moerman T, et al. SCENIC: single-cell regulatory network inference and clustering. *Nat Methods*. 2017;14:1083-1086.

13. Qiu X, Hill A, Packer J, Lin D, Ma YA, Trapnell C. Single-cell mRNA quantification and differential analysis with Census. *Nat Methods*. 2017;14:309-315.

14. Efremova M, Vento-Tormo M, Teichmann SA, Vento-Tormo R. CellPhoneDB: inferring cell-cell communication from combined expression of multi-subunit ligand-receptor complexes. *Nat Protoc*. 2020;15:1484-1506.
