# Supplementary figures and images for: Single‐cell sequencing of mouse heart cellular heterogeneity in hypercholesterolemia reveals the mechanism of myocardial damage
Source: Clin Transl Med. 2022 Jul 20;12(7):e951. doi: 10.1002/ctm2.951 (PMC9301086; doi:10.1002/ctm2.951)

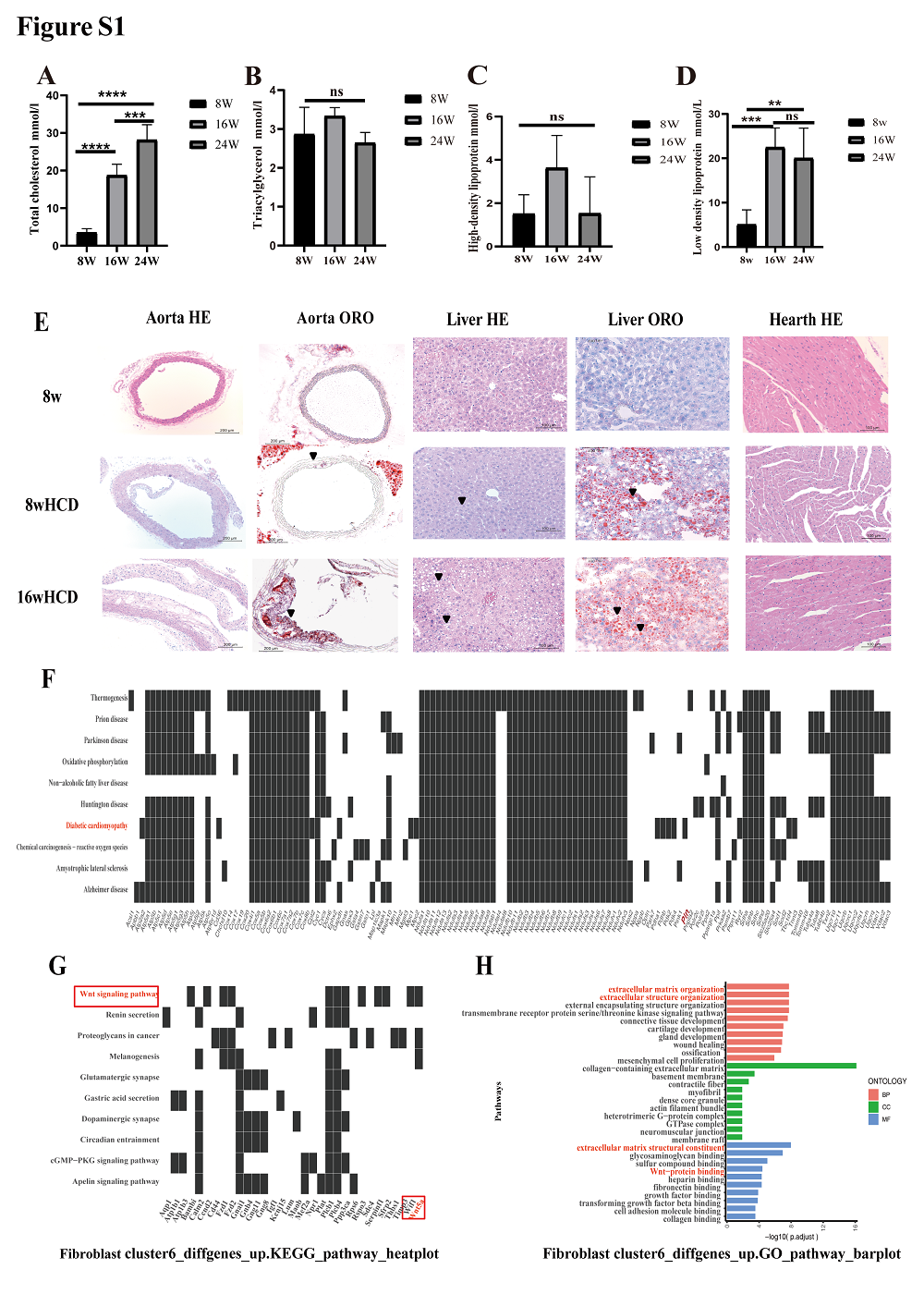

Supplement: Supplementary file 2 — Figure S1 [file CTM2-12-e951-s020.tif]

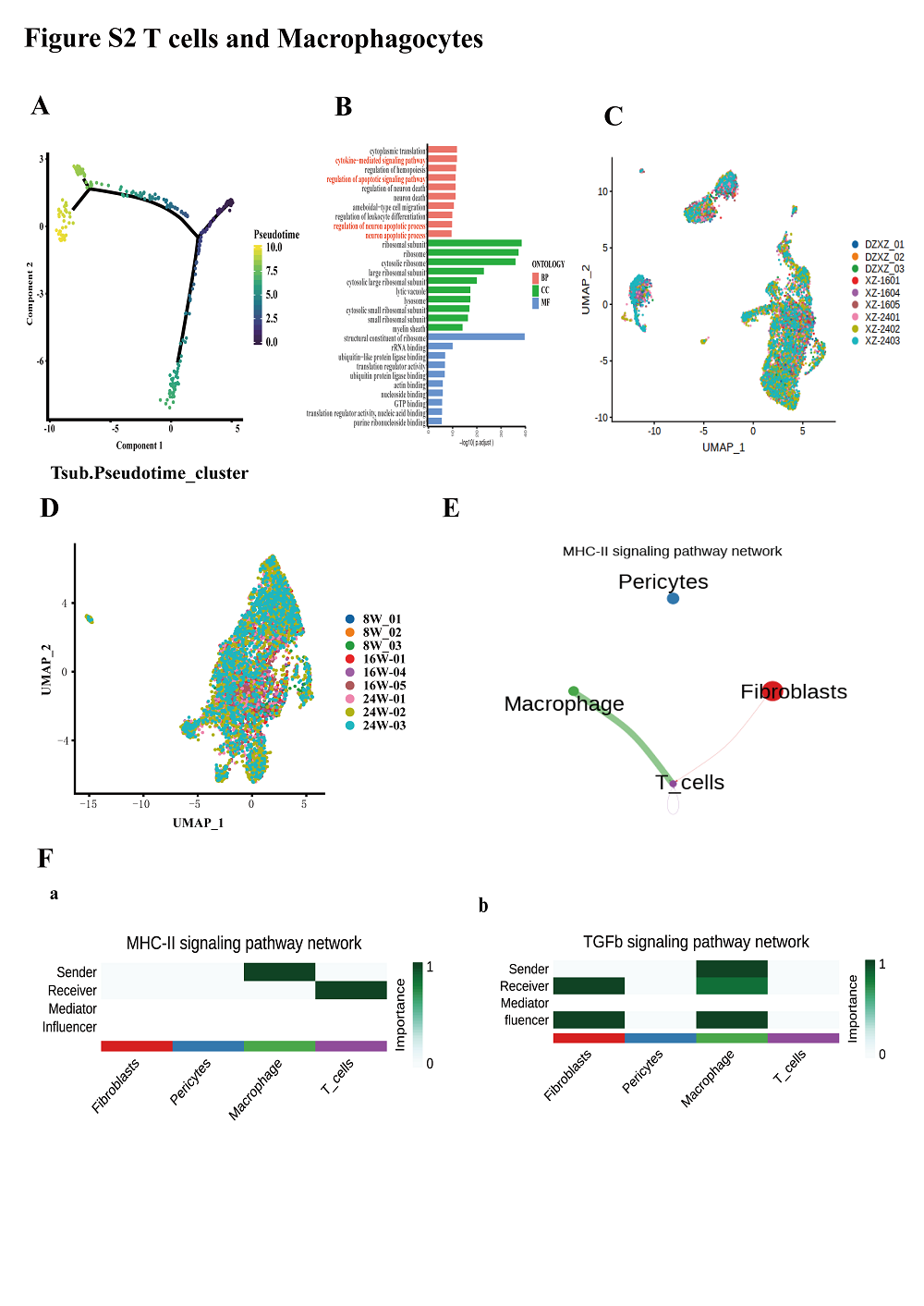

Supplement: Supplementary file 3 — Figure S2 [file CTM2-12-e951-s010.tif]

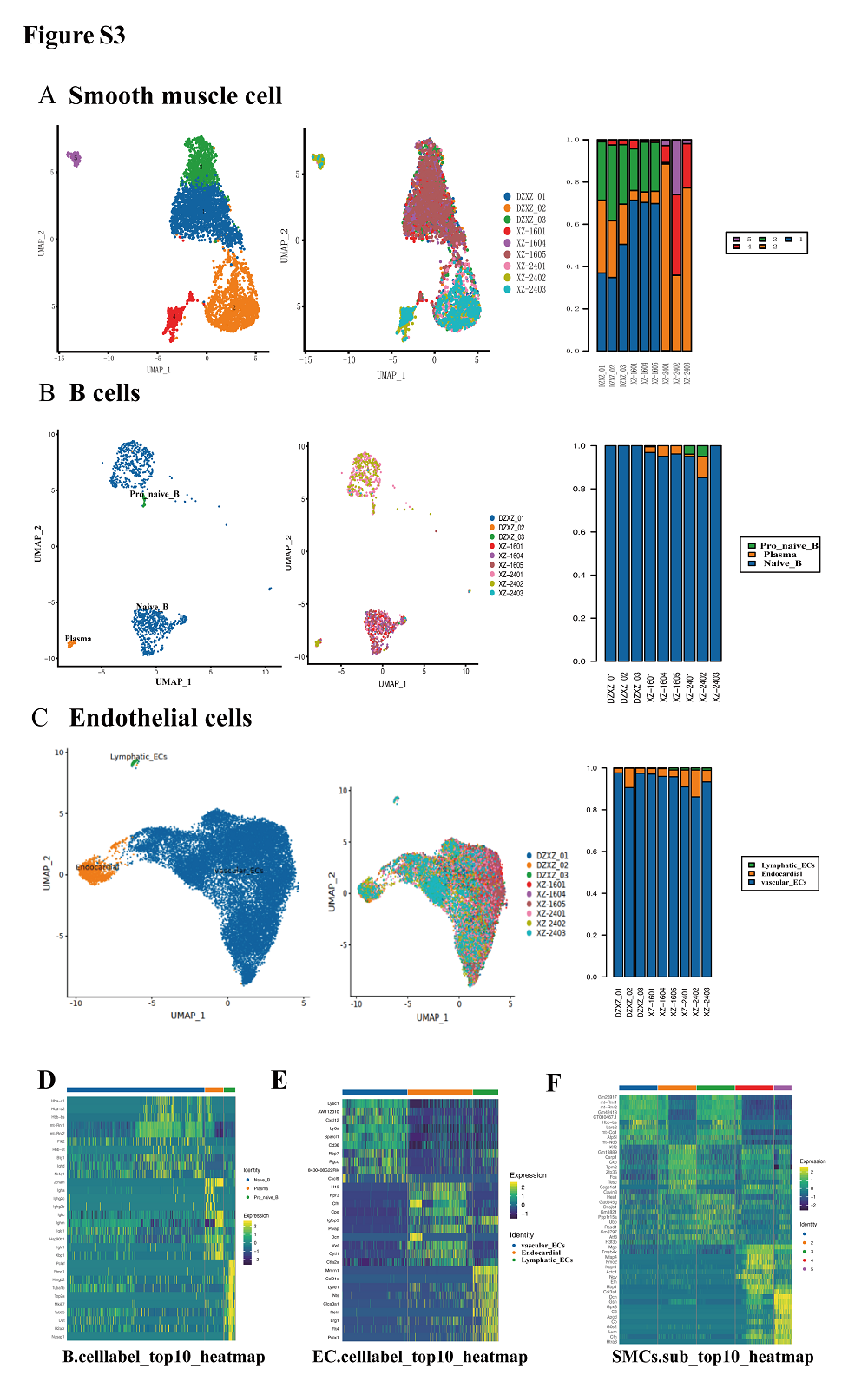

Supplement: Supplementary file 4 — Figure S3 [file CTM2-12-e951-s007.tif]
